# Tracing PRX1^+^ cells during molar formation and periodontal ligament reconstruction

**DOI:** 10.1038/s41368-021-00155-z

**Published:** 2022-01-25

**Authors:** Xuyan Gong, Han Zhang, Xiaoqiao Xu, Yunpeng Ding, Xingbo Yang, Zhiyang Cheng, Dike Tao, Congjiao Hu, Yaozu Xiang, Yao Sun

**Affiliations:** 1 Department of Implantology, School & Hospital of Stomatology, Tongji University, Shanghai, China; 2 Shanghai Engineering Research Center of Tooth Restoration and Regeneration, Shanghai, China; 3 Shanghai East Hospital, School of Life Sciences and Technology, Tongji University, Shanghai, China

**Keywords:** Mesenchymal stem cells, Cell lineage

## Abstract

Neural crest-derived mesenchymal stem cells (MSCs) are known to play an essential function during tooth and skeletal development. PRX1^+^ cells constitute an important MSC subtype that is implicated in osteogenesis. However, their potential function in tooth development and regeneration remains elusive. In the present study, we first assessed the cell fate of PRX1^+^ cells during molar development and periodontal ligament (PDL) formation in mice. Furthermore, single-cell RNA sequencing analysis was performed to study the distribution of PRX1^+^ cells in PDL cells. The behavior of PRX1^+^ cells during PDL reconstruction was investigated using an allogeneic transplanted tooth model. Although PRX1^+^ cells are spatial specific and can differentiate into almost all types of mesenchymal cells in first molars, their distribution in third molars is highly limited. The PDL formation is associated with a high number of PRX1^+^ cells; during transplanted teeth PDL reconstruction, PRX1^+^ cells from the recipient alveolar bone participate in angiogenesis as pericytes. Overall, PRX1^+^ cells are a key subtype of dental MSCs involved in the formation of mouse molar and PDL and participate in angiogenesis as pericytes during PDL reconstruction after tooth transplantation.

## Introduction

The cranial neural crest-derived mesenchymal stem cells (MSCs) regulate multiple events of the tooth development process.^1–4^ Although a variety of MSC subtypes are involved in tooth development, the mechanism underlying their specific participation during molar development and remodeling of periodontal tissues needs to be investigated.^5^ Recently, genetic Cre-mediated lineage-tracing studies have revealed diverse populations of MSCs and differentiated functional cells.^6,7^ It is believed that lineage-tracing studies about MSC functions will open a new perspective to investigate tooth development and regeneration.

The paired-related homeobox gene-1 (*Prx1*) is a transcription factor that is widely expressed in the limb bud mesenchyme and constitutes a subset of craniofacial mesenchyme.^8–10^ PRX1^+^ cells are MSCs that are extensively involved in the development of limb bones and craniofacial mesenchyme from the early embryonic stage.^11–14^ In addition, PRX1^+^ cells participate in the repair and regeneration of tissues; PRX1^+^ cells and their progeny are responsible for the regeneration of calvarial bones.^15^ During a fracture, perivascular PRX1^+^ cells elicit a periosteal and endosteal response,^16^ which could be a major source of osteoblasts and chondrocytes in the fractured callus.^17^ A recent study reported that *Prx1* is involved in the periodontal regeneration of mouse incisors.^18^ Meanwhile, *Prx1* is essential for the development and maintenance of the blood.^19^ Thus, the function of PRX1^+^ cells in tissue regeneration and repair could be related to angiogenesis.^20^

The development of molars significantly differs from incisors in mouse^21^ and is more similar to that of humans. However, the function of PRX1^+^ cells in molar development and regeneration of molar periodontal ligament (PDL) remains elusive. In the present study, we investigated the potential function of PRX1^+^ cells in molar development and PDL tissue regeneration by exploring the fates of PRX1^+^ cells during molar development using a lineage-tracing mouse model and single-cell sequencing (scRNA-seq). In addition, we defined the functions of PRX1^+^ cells in PDL reconstruction using an allograft tooth model.

## Results

### Distribution of PRX1^+^ cells during early tooth development

**Fig. 1:**
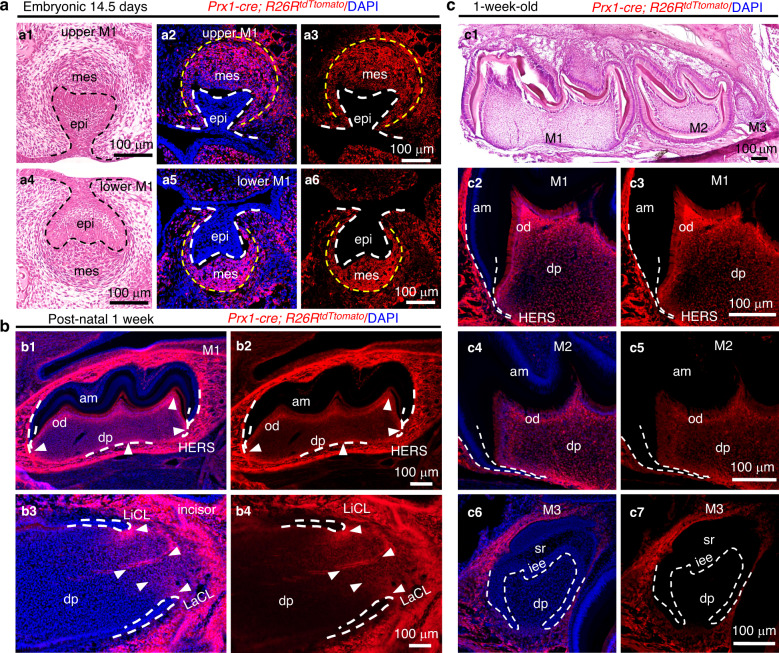
Distribution of PRX1^+^ cells in tooth germ, incisors, and molars. **a** H&E staining (**a1**, **a4**) and distribution of PRX1^+^ cells in the tooth germ of first molar of the maxillary (**a2**, **a3**) and mandible (**a5**, **a6**) at 14.5 days of the embryo (E 14.5). The edge of the tooth germ epithelium is outlined with a dotted line. PRX1^+^ cells are concentrated in the mesenchyme of the tooth germ and are completely absent in the epithelium (a2, a3, a5, and a6) (*n* = 4). **b** Distribution of PRX1^+^ cells in M1 and incisor of mice at 1 week. White arrowheads: high concentrations of PRX1^+^ cells in the pulp cavity. In M1, PRX1^+^ cells appear in the pulp cavity, especially in odontoblasts (**b1**, **b2**). In incisor, they are primarily present in the stem cell niche area near the buccal and lingual cervical loop (**b3**, **b4**). In addition, high concentrations are present in the alveolar bone around M1 and incisor (*n* = 4). **c** Comparison of distribution patterns of PRX1^+^ cells in M1-M3 of one-week-old mice. **c1** H&E staining of M1-M3. Numerous PRX1^+^ cells expressing tdTomato fluorescence are distributed in the pulp cavity of M1 (**c2**, **c3**) and the number of positive cells decreases in M2 (**c4**, **c5**). However, it is not observed in the dental papilla of M3 (**c6**, **c7**) (*n* = 4). epi epithelium, mes mesenchyme, od odontoblast, am ameloblast, dp dental pulp, HERS Hertwig’s epithelial root sheath, laCL labial cervical loop, liCL lingual cervical loop, sr stellate reticulum, iee inner enamel epithelium. Scale bar: 100 μm

In mice, molar development initiates at around the embryonic day (E) 11.5. *Prx1* is expressed in the mesenchyme before E11.5.^10,22^ We used a transgenic mouse model—*Prx1-cre*; *R26R*^*tdTomato*^, PRX1^+^ cells labeled with tdTomato (in red) to observe the distribution of PRX1^+^ cells in molars and incisors. Prx1-expressing cells and their progeny (all expressing tdTomato) are primarily located within the mesenchyme space of the first molar, including both the cap stage of morphogenesis (Fig. 1a) and the cyto-differentiation stage (Fig. 1b1–b2). No difference was observed in the distribution of PRX1^+^ cells in M1 (the first molar) of the upper (Fig. 1a1–a3) and lower jaws (Fig. 1a4–a6). In addition, we studied whether the positive cells in different molars had the same distribution pattern. PRX1^+^ cells were primarily distributed in the pulp cavity of M1, with a reduced distribution in M2. There were almost no positive cells in M3 (Fig. 1c).

A stem cell niche exists at the root of the mouse incisor which is continuously renewed throughout life.^23^ We observed the incisors of 1-week-old mice and found that PRX1^+^ cells appeared in the stem cell niche between the labial and lingual cervical loop. However, it was difficult to observe in the pulp of incisor (Fig. 1b3–4).

### Differentiation of PRX1^+^ cells during root formation

**Fig. 2:**
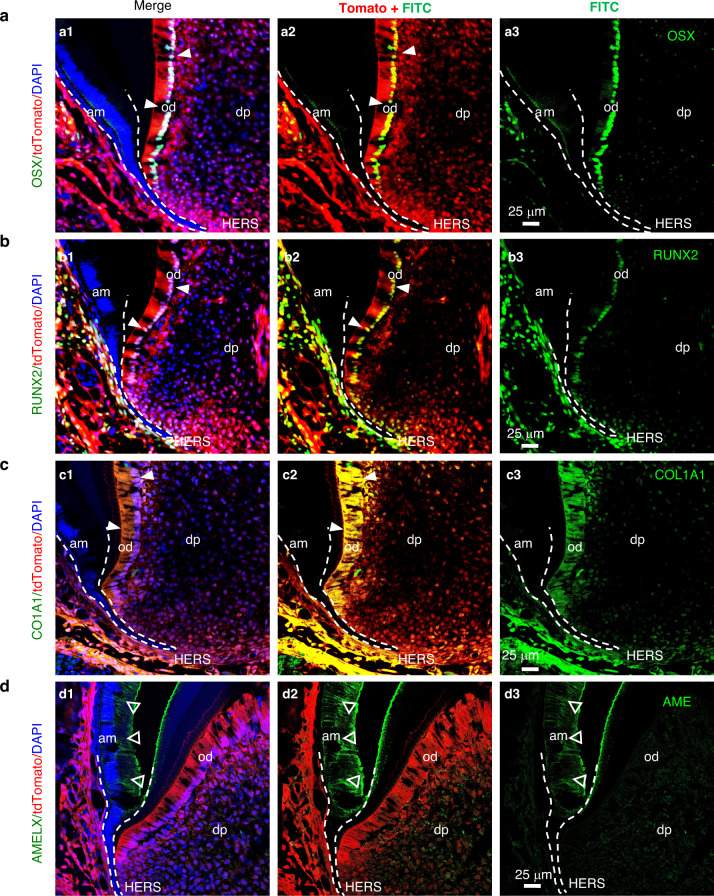
Lineage tracing analysis of PRX1+ cell progeny during molar root formation. PRX1^+^ cells are co-expressed with mesenchymal progenitor cell markers and odontogenic lineage markers. **a**–**d** Co-staining of PRX1^+^ cells with the pre-odontoblast cell marker OSX (**a**), RUNX2 (**b**), COL1A1 (**c**), and the marker of ameloblast AMELX (**d**). In (**a**–**c**), white arrowheads point to the places where red (PRX1^+^) and green cells overlap; In (**d**), the white hollow arrowheads point to the place where red and green do not overlap; *n* = 4; Scale bar: 25 μm

We next checked the differentiation of these cells in cyto-differentiation stage. RUNX2 is required for the differentiation of multipotential mesenchymal progenitor cells into preosteoblasts/preodontoblasts.^24^ OSX is required at a later maturation stage of preosteoblasts/preodontoblasts into functional osteoblasts/odontoblasts.^25^ Both these proteins are strongly co-expressed with PRX1^+^ cells (Fig. 2a, b). Although PRX1^+^ cells are also co-expressed with odontoblast marker COL1A1 (Fig. 2c), these are not co-localized with epithelial-derived ameloblasts (Fig. 2d). Overall, PRX1^+^ cells differentiate into odontoblasts and dental pulp cells during odontogenesis.

### Identification of PRX1-expressing cells in adult human molars

**Fig. 3:**
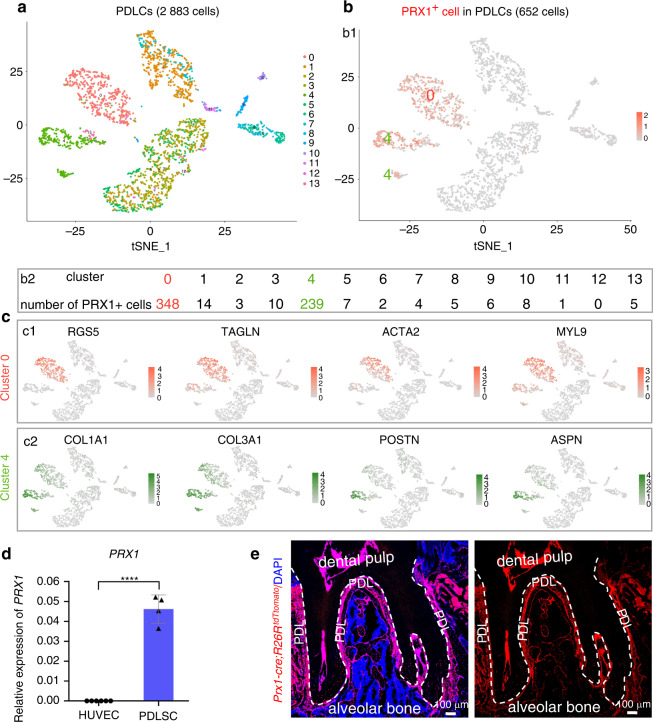
scRNA-seq analysis of PRX1-expressing cells in human PDL. **a** tSNE visualization of color-coded clustering of PDLCs (*n* = 2883 cells). **b** Distribution of PRX1-expressing cells in PDLCs, primarily in clusters 0 and 4. **b1** is the visualization of PRX1-expressing cells, and (**b2**) refers to the number of PRX1-expressing cells in all clusters. The number of PRX1-expressing cells is considerably higher in cluster 0 and cluster 4 than in other clusters. **c** Characterization of clusters 0 and 4 (PRX1-expressing cells). **c1**: Cluster 0 expresses MYL9, a marker of MSC, and RGS5, TAGLN, ACTA2, markers of pericytes, whereas cluster 4 expresses the fibroblast markers, collagen proteins (COL1A1 and COL3A1), and PDL specific extracellular matrix proteins POSTN and ASPN (**c2**). **d** Expression of PRX1 in human PDLSC and HUVEC in vitro. PDLSC highly expresses PRX1, while HUVEC hardly expresses PRX1 (*n* = 4–6). ****P < 0.000 1. **e** Distribution of PRX1^+^ cells in M1 of adult mice. PRX1^+^ cells are abundantly distributed in the PDL and pulp cavity (*n* = 4)

ScRNA-seq technology has been used in dental research to reveal the atlas of mouse tooth^26,27^ and explore the functions of a subtype of cells. Based on a recent study of single-cell atlas of human teeth,^28^ we explored the cell distribution pattern of PRX1^+^ cells in human molars. We found that PRX1^+^ cells occupy a high proportion of the third molar periodontal cells (652/2,883) (Fig. 3a, b). Further analysis showed that PRX1-expressing cells were primarily distributed in clusters 0 and 4 (Fig. 3b). Cluster 0 expresses MSC markers, and also highly expresses perivascular markers, such as TAGLN (Fig. 3c1), which is ubiquitously expressed in vascular and visceral smooth muscle, and is an early marker of smooth muscle differentiation. In other words, PRX1-expressing cells in PDLCs highly overlap with the population of perivascular cells. Cluster 4 is a fibroblast population that secretes broad-spectrum collagen proteins COL1A1 and COL3A1, as well as extracellular matrix proteins, namely ASPN and POSTN that are specifically expressed in the PDL (Fig. 3c2). ASPN and POSTN are markers of mature PDL and are preferentially expressed in PDL.^29,30^ In addition to scRNA-seq data, we also detected the expression of Prx1 in human PDLSC and angiogenesis-related cells HUVEC cultured in vitro. High expression of PRX1 was also detected in human PDLSC, but not in HUVEC (Fig. 3d).

### Tracing PRX1^+^ cells in mouse PDL

**Fig. 4:**
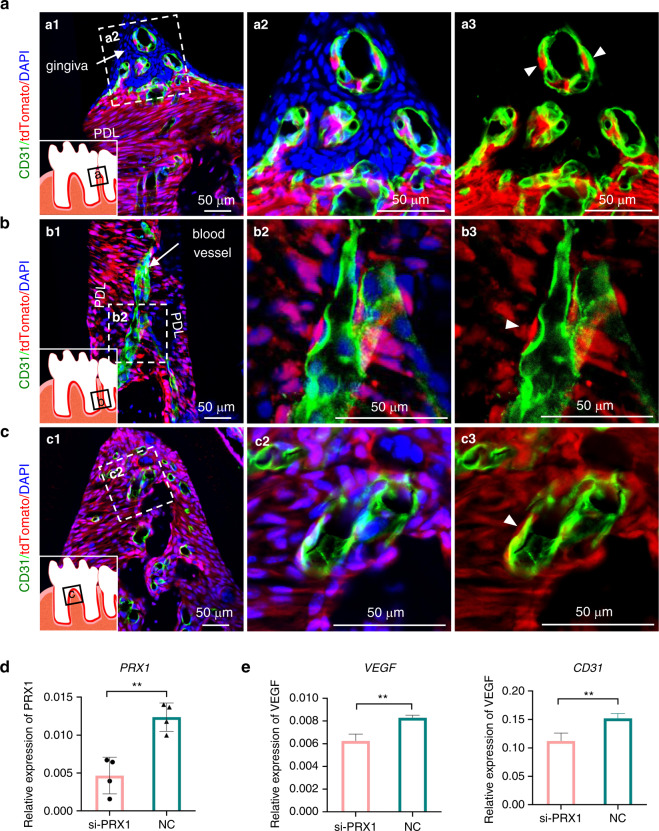
Distribution of PRX1^+^ cells in the PDL of adult mouse molars and its function in angiogenesis. **a**–**c** In the PDL of *Prx1-cre; R26R*^*tdTomato*^ mice, the relationship between PRX1^+^ cells and vascular endothelial cells in three parts of the periodontal: the upper half of the root (**a**), the lower half of the root (**b**), and the alveolar crest (**c**). PRX1^+^ cells (white arrowheads) are surrounding the blood vessels (*n* = 6). **d** Knock down of Prx1 in PDLSCs. **P < 0.01. **e** Co-cultur**e** of HUVECs and PDLSCs with down-regulation of *Prx1*, test the expression of CD31 and VEGF (*n* = 4). **P < 0.01

The staining and scRNA-seq analysis revealed that PRX1^+^ cells were distributed in large numbers in PDLCs and overlapped with perivascular cells. We further tested PRX1^+^ cells in the molar PDL of adult *Prx1-cre; R26R*^*tdTomato*^ mice. The PDL of adult mouse molars contains a high number of PRX1^+^ cells (Fig. 3e). The relationship between PRX1^+^ cells and blood vessels in PDL was shown by co-staining of tdTomato with endothelial cell markers CD31. We observed the relationship between PRX1^+^ cells and blood vessels in three areas of the periodontal: the upper half of the root (Fig. 4a), the lower half of the root (Fig. 4b), and the alveolar crest (Fig. 4c). The results showed that blood vessels were often accompanied by PRX1^+^ cells in molar PDL (Fig. 4a–c). Since most of the cells in the gingival are of epithelial origin (not PRX1^+^ cells), this phenomenon is extremely obvious: newly migrating PRX1^+^ cells appeared around vascular endothelial cells (Fig. 4a).

Based on the finding that Prx1^+^ cells are involved in the angiogenesis of PDLCs, we wanted to study whether the absence of *Prx1* changed the angiogenesis ability of PDLCs. Therefore, we knocked down *Prx1* in PDLSCs (Fig. 4d), and co-cultured the PDLSCs with HUVECs. A decline in CD31 and VEGF was detected (Fig. 4e).

### Establishment of allograft tooth transplantation model

**Fig. 5:**
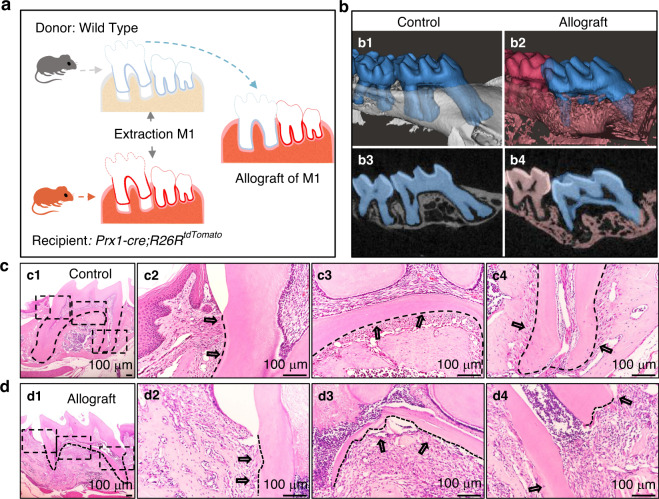
Establishment of allograft tooth transplantation model. **a** Schematic diagram of the model. **b** Micro-CT image of the control group (b1, b3) and the allograft group (b2, b4). The roots of the allograft group showed tooth absorption (b2, b4). **c**, **d** Representative H&E staining of control (**c**) and allograft group (d). During the restoration of teeth after transplantation, the PDL partially reattached to the cementum surface (d2), part of the samples accompanied by pulp necrosis and root resorption (**d3**, **d4**). The black arrows point to the PDL near the crown in **c2** and **d2**, the PDL at the root furcation in **c3** and **d3**, and the PDL of the root apex in **c4** and **d4**. **c2**-**c4** are magnified views of the c1 dotted areas, **d2-d4** are magnified views of the **d1** dotted areas

To study whether PRX1^+^ cells could participate in the regeneration of molar PDL, we established an allograft model and explored the functions of PRX1^+^ cells in PDL repair and regeneration of molars. The process of construction of the model was shown in Fig. 5a, namely transplanting the molars of wild-type (WT) mice into *Prx1-cre; R26R*^*tdTomato*^ mice. Two weeks after surgery, nearly half of the mice (10/18) recovered well, and the other half showed root resorption (8/18). Micro-CT analysis (Fig. 5b) and H&E staining showed root resorption in the allograft group (Fig. 5d).

### PRX1^+^ cells are involved in angiogenesis during periodontal ligament reconstruction

**Fig. 6:**
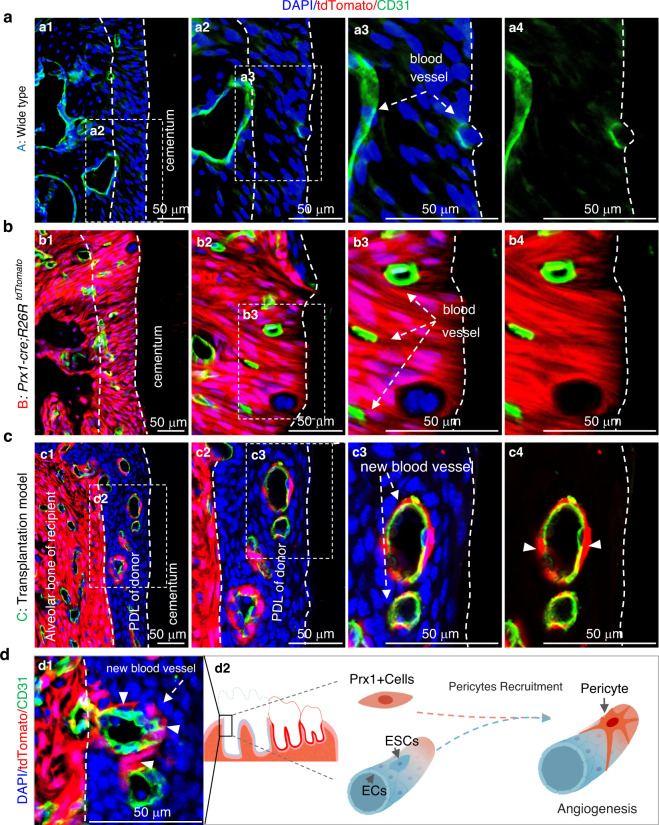
PRX1^+^ cells in the reconstruction of PDL. **a**–**c** PDL and blood vessels of WT mice (**a**), *Prx1-cre; R26R*^*tdTtomato*^ mice (**b**), tooth replantation model mice (**c**), respectively. **c,d** Recipient PRX1^+^ cells (labeled in red) migrated to the PDL of the implanted WT M1 to form new blood vessels, and white arrows in c3 and d1 point to the new angiogenesis in WT PDL, white arrowheads point to migrated PRX1^+^ cells. **d2**, Schematic diagram of PRX1^+^ cells involved in angiogenesis as pericyte during the restoration of PDL. *n* = 18 in tooth transplantation model (including Fig. 5 and Fig. 6)

In order to compare and observe the migration of PRX1^+^ cells in tooth transplantation model, we showed three models at the same time (model A, B, C). In wild-type mice (model A), we can only observe CD31-labeled (green) blood vessels in PDL (Fig. 6a). In *Prx1-cre; R26R*^*tdTomato*^ mice (model B), the PDL was almost all marked in red (except for the epithelial cell rests of Malassez), as shown in Fig. 6b and Fig. 4a–c. In the allograft tooth transplantation model (model C), vibrant endothelial cells from the recipient mice migrated to the damaged PDL to restore the nutrient supply (Fig. 6c). In the allogeneic tooth graft model, PRX1^+^ cells from recipient mice were labeled in red, and cells from donor mice were without fluorescence. Accompanied by the migration of vascular endothelial cells into the PDL of the implanted WT M1, a substantial migration of PRX1^+^ cells to the recovered PDL was observed. These migrating PRX1^+^ cells were actively involved in the angiogenesis during PDL reconstruction (Fig. 6c, d). The role of PRX1^+^ cells as pericyte during PDL reconstruction was shown in Fig. 6d, in the form of immunofluorescence picture (Fig. 6d1) and schematic diagram (Fig. 6d2).

## Discussion

Recently, *Prx1* has been used to label MSCs during the development of bones and teeth. However, the exact function of PRX1^+^ cells in tooth development has remained unclear. Therefore, the *Prx1-cre; R26R*^*tdTomato*^ reporter mice were used to study the distribution pattern and fate of PRX1^+^ cells during the organogenesis of molar and PDL formation. Our results showed that PRX1^+^ cells and their progeny occupied most of the first molar mesenchyme, starting from dental papilla at the cap stage. PRX1^+^ cells can differentiate into the majority of cell types in M1, including odontoblast progenitor cells, odontoblasts, fibroblasts, and dental pulp cells in cytodifferentiation stages.

It is not clear if the development mechanism of each molar is the same. In general, all molars of mice share a similar gene expression pattern during development.^31^ Therefore, stem cells originating from these molars are similar. We reported that MSCs subtypes between molars could be different. The distribution of PRX1^+^ cells varied significantly between the mandibular first, second, and third molars, especially if M1 is compared to the M3. Although PRX1^+^ cells are rich in the mesenchymal of M1, only a few PRX1^+^ cells were observed even in the bell-stage papillae of M3. This could be attributed to different developmental time points between M1 and M3. M1 starts at the embryonic stage and completes crown morphology at birth, whereas M3 only begins to develop after birth. There exists a difference in the types of MSCs in the embryonic period and after birth.^32,33^ The results indicate the heterogeneity of MSCs of molars, a finding similar to that reported recently in bone development, which stated that bone formation before and after adolescence is controlled by distinct progenitors.^34^ In addition, the heterogeneity of MSCs is related to the location of their origin.^35,36^ MSCs in different mouse molars contain different concentrations of key tooth development signaling molecules, i.e., bone morphogenetic proteins (BMPs), or respond differently to sonic hedgehog (Shh). These differences, in turn, lead to varying MSCs behaviors, eventually resulting in teeth with different crown morphologies and root numbers. The findings on PRX1^+^ cell distribution provide insights into further understanding of the dental development rhythm and the gene expression characteristics among molars.

In addition to murine lineage-tracing approaches, scRNA-seq technology has greatly contributed to the study of characteristics of certain dental cell subgroups in recent years.^37,38^ The scRNA-seq analysis of periodontal ligament cells has revealed a subgroup of PRX1^+^ cells that express markers of perivascular cells, which is consistent with the pro-angiogenic function of PRX1^+^ cells reported in the previous literature.^39^ For example, *Prx1* promotes angiogenic differentiation during the development of the pituitary gland.^40^ In addition, MSC is a major constituent of the hematopoietic stem cell (HSC) niche and a source of perivascular cells.^41^ Prx1-expressing cells constitute a classic MSC subpopulation, which is involved in angiogenesis during organ development and tissue repair.^42^ In addition, PRX1^+^ cells participate in cranio-maxillofacial tissue regeneration and damage repair. It is reported that *Prx1* contributes to the regeneration of periodontal tissue of mouse incisors.^15,17,18^ To investigate the specific function of PRX1^+^ cells in mouse molar PDL remodeling and regeneration, we used WT mice as donors and *Prx1-cre; R26R*^*tdTomato*^ mice as recipients and established an allograft tooth transplantation model. Mouse M1 highly expresses PRX1 (Fig. 3e and Fig. 4a–c), so we chose mouse M1 for tooth transplantation experiments. The PRX1^+^ cells in the recipient mice were marked with red fluorescence; the red cells from the recipient actively migrated to the donor WT M1’s PDL, always accompanied by the distribution of new blood vessels in PDL. This is new evidence that confirms that PRX1 + cells are involved in angiogenesis during PDL repair, which could provide a new molecular target for regulating blood vessel regeneration in PDL. Next, we implanted the teeth of *Prx1-cre; R26R*^*tdTomato*^ mice in WT recipient mice; however, we found that the PRX1^+^ cells in residual donor PDL did not migrate to WT mice (data not shown), which could be related to the source and activity of PDLSCs. PDLSCs derived from the alveolar bone had a higher proliferative ability and stronger differentiation potential than PDLSCs derived from the conventional tooth root surface.^43^ It is worth noting that the root canal therapy of replanted teeth may also affect the vitality of PDLSCs. In the future, root canal therapy of the donor tooth before transplantation should be considered to prevent severe inflammation, and whether it could improve the vitality of the implanted donor PDLCs remains to be explored. In future studies, *Prx1* could be knocked out in the allograft model to further study how PRX1^+^ cells participate in angiogenesis during PDL tissue repair.

In conclusion, PRX1^+^ cells are involved in the development of M1 and the differentiation of almost all mesenchymal cell types required for M1 development. The distribution of PRX1^+^ cells between molars varies and is of great significance in our understanding of the temporal development characteristics of M1–M3 molars in mice. In addition, PRX1^+^ cells overlap with perivascular cells in the PDLCs of adult molars, which are involved in the angiogenesis in PDL development and repair. These findings provide new clues to understand the significance of Prx1, an important stem cell subtype, in molar development and regeneration.

## Materials and methods

### Animals

The transgenic mouse lines studied included *Prx1-cre* mice (targeting MSC progenitors), and *Rosa26*^*LoxP-STOP-loxP-tdTomato*^; these were purchased from Cyagen (Beijing, China). The *Prx1-cre* mice were crossed with *R26R*^*tdTomato*^ mice (*Prx1-cre; R26R*^*tdTomato*^ mice) and the expressing PRX1 and their progeny emitted red fluorescence. The proliferation, differentiation, and migration of Prx1^+^ cells were tracked. Animals were maintained in a specific pathogen-free (SPF) facility under a 12:12-h day/night illumination cycle. Animals were euthanized by cervical dislocation after inhalation anesthesia. The Animal Welfare Committee of the Affiliated Stomatology Hospital of Tongji University (2019-DW-040) approved all animal experimental protocols used on mice.

### Re-analysis of scRNA-seq data

The scRNA-seq data were obtained from the GEO database (GSE161267).^28^ The analysis was performed using Seurat v4.0.5 and R version 4.0.4. Clusters were visualized using t-Distributed Stochastic Neighbor Embedding (tSNE). Data were scaled and transformed using sctransform_0.3.2 for variance stabilization. Any subsequent analysis was done using raw data and not data transformed after integration.

### Establishment of the allograft tooth model

First, the right maxillary first molars of 6-week-old WT mice were extracted, and were placed in normal saline after being rinsed gently. Next, the upper first molars of the 6-week-old *Prx1-cre; R26R*^*tdTomato*^ mice were extracted, and after ensuring that no roots remained, the first molars of the previously prepared WT mice were implanted immediately. The mice were fed soft food after the operation. Samples were sacrificed after two weeks. A total of 18 mice were used in the allogeneic tooth replantation models in Figs. 5 and 6.

### Immunofluorescence and image acquisition

Maxillary or mandibular bones of mice were decalcified in 10% ethylene diamine tetraacetic acid (EDTA) (pH 7.4) at 4 °C for 21 d. For immunofluorescence staining, specimens were embedded in optimal cutting temperature compound (OCT), and sectioned into 10-μm thick sections. Sections were treated with 3% hydrogen peroxide and goat serum blocking, and then they were incubated with a primary antibody. The following primary antibodies were used: anti-Osterix (1:300; Abcam, Cambridge, UK), anti-RUNX2 (1:100; Abcam), anti-type I collagen (1:200; Boster Biological Technology, Wuhan, China), anti-Amelogenin (1:100; Santa Cruz Biotechnology, Dallas, TX), anti-CD31 (1:100; Affinity Biosciences, Changzhou, China). Sections were subsequently incubated by Alexa Fluor 488 IgG (1:1000; Invitrogen) and/or Alexa Fluor 568 IgG (1:1000; Invitrogen), and counterstained with DAPI (Sigma-Aldrich).

The images were captured using a confocal microscope (Nikon, TI2-E + A1 R, Japan), and processed using the ImageJ software (US National Institutes of Health, United States).

### Microcomputed tomography (micro-CT) analysis

Alveolar bones with teeth dissected from mice were fixed in 4% paraformaldehyde (PFA) for 48 h. Use micro-CT (μCT50, Scanco Medical, Zurich, Switzerland) for tissue tomography and output in DICOM format. Image data were reconstructed and analyzed using the Mimics 13.0 software.

### Isolation, culture, and transfection of human PDLSCs

Normal impacted third molars (*n* = 8) were collected from five individuals aged 18–25 years at the Department of Oral and Maxillofacial Surgery, School & Hospital of Stomatology, Tongji University, Shanghai, China. The use of human tissue for research was approved by the Ethics Committee at the Affiliated Stomatology Hospital of Tongji University. Human PDLSCs were freshly isolated according to previously reported protocols^44,45^ and cultured in alpha-modification of Eagle’s medium (α-MEM, HyClone, USA) containing 10% fetal bovine serum (FBS, Gibco, USA), 100 units per mL penicillin–streptomycin (HyClone), and 100 μmol·L^−1^ ascorbic acid (Sigma–Aldrich). Cells at passages P3–P5 were used for cytological experiments

PEI transfection reagent (proteintech, Wuhan, China) was used for the transfection of siRNAs. Sequences of siRNAs used were:

GAAUAGGACAACCUUCAAUTT (5′−3′) and AUUGAAGGUUGUCCUAUUCTT (5′−3′).

### RNA extraction and real-time quantitative polymerase chain reaction

The co-cultured PDLSCs and ECs were crushed into Trizol reagent (Invitrogen) according to the manufacturer’s protocol. First-stand complementary DNA (cDNA) was synthesized using a Transcriptor First Strand cDNA Synthesis Kit (Roche, Basel, Switzerland). The primer sequences were:

Prx1: 5′-CACCTGCTAGACCTGGAGGAA-3′ and 5′-GCTGCTATTGAAGGTTGTCCTATT-3′; β-actin: 5′-AGGGCATACCCCTCGTAGAT-3′ and 5′-ACGTTGCTATCCAGGCTGTG-3′; CD31: 5′-TGTCAAGTAAGGTGGTGGAGTCT-3′ and 5′-AGGCGTGGTTGGCTCTGTT-3′; Vegf: 5′-CCCACTGAGGAGTCCAACAT-3′ and 5′-AAATGCTTTCTCCGCTCTGA-3′.

### Statistical analysis

All data were performed by SPSS 20.0 and GraphPad Prism 8.0. Comparisons between two groups were analyzed by Student’s *t-*test. *P* < 0.05 was considered statistically significant.

## Data Availability

The data used and/or analyzed during the current study are contained within the manuscript or available from the corresponding author on reasonable request.
